# Provincial Maternal Mortality Surveillance Systems in China

**DOI:** 10.1155/2014/187896

**Published:** 2014-06-03

**Authors:** Xiao-Ling Gan, Chang-Lai Hao, Xiao-Jing Dong, Sophie Alexander, Michèle Wilmet Dramaix, Li-Na Hu, Wei-Hong Zhang

**Affiliations:** ^1^Obstetrics and Gynecology Department, The Second Affiliated Hospital of Chongqing Medical University, Chongqing 400016, China; ^2^School of Public Health, Epidemiology, Biostatistics and Clinical Research Centre, Université Libre de Bruxelles (ULB), Brussels 1070, Belgium; ^3^Hematology Department, The Affiliated Hospital of Chengde Medical College, Chengde 067000, China

## Abstract

*Background*. Provincial maternal mortality surveillance systems (PMMSS) have been set up in nearly all the provinces in China to monitor local maternal mortality and provide the evidence for maternal health interventions suited to local conditions. However, till now little is known outside of China about the characteristics of PMMSS. *Methods*. A systematic review of the literature contained in PubMed and China Academic Journal Network Publishing database was carried out. The current situation on PMMSS was described. Provincial disparities on PMMR in six provinces were analyzed by Poisson regression analysis. *Results*. A total of 35 studies met the inclusion criteria, of which 31 were published in Chinese. PMMSS were set up and adjusted by the provincial government based on their own financial resources and demand. Provinces from remote region had the highest risk of maternal mortality, followed by provinces from inland region and coast region. *Conclusions*. PMMSS may be the most reliable data source for measuring provincial level MMR in each province. Great provincial disparities on PMMSS and PMMR do exist within the country; more emphasis should be placed on improving PMMSS and reducing PMMR particularly in the provinces with high maternal death burden.

## 1. Introduction

China endorsed the United Nations Millennium Development Goals (MDGs) in 2000, which include a reduction of the maternal mortality ratio (MMR) by 2015 from 89 maternal deaths per 100 000 live births in 1990 to 22/100 000. During the past ten years, China has placed great emphasis on the maternal health, and MMR in China has declined in some degree [1, 2]. China's national MMR in 2010 was 30.0/100,000, a decrease of 43.4% from 2000 [3]. However, the progress has in general been slow and shown obvious regional disparities [4, 5]. With the target date for achieving the MDG-5 only 2 years away, the question of how to accelerate and even the decline has become more pressing. Reliable information about provincial maternal mortality in China is essential for knowing where the risk is the highest as well as where the numbers of deaths are the greatest within the country and more importantly for resource mobilization to reduce MMR and assessment of progress towards achieving MDG.

National Maternal and Child Mortality Surveillance System (NMCMSS), representing all 31 provinces and covering 60 million people, has been the principal data source for estimating national MMR in China [1, 2]. Nevertheless, despite the current utility of the NMCMSS for generating evidence for national health policy, considering the big population in China, the generalizability of the NMCMSS data has been called into question since the system covers only a small fraction of the population [6, 7]. The National Maternal and Child Health Routine Reporting System (NMCHRS) is another source of maternal mortality data which theoretically covers the whole population in China [8]. However, the underreporting in the routine reporting system and lack of details in causes of maternal death limit its use [1, 9].

**Figure 1:**
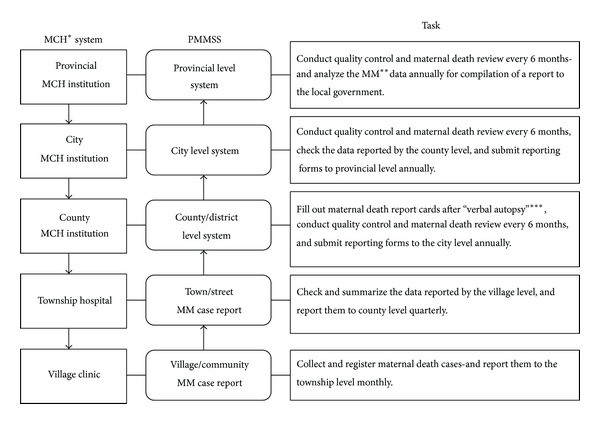
The common structure and operation procedure of provincial maternal mortality surveillance system (PMMSS) in China. *MCH: maternal and child health; **MM: maternal mortality; ***verbal autopsy: investigation on the circumstances, timing, and cause of maternal death was conducted by at least one specialty trained obstetrician through document reviewing or personal interviewing.

Given the size and diversity of China, reducing maternal deaths in the whole of China under the same policies is a great challenge in particular. In order to monitor local maternal mortality levels and to provide scientific evidence for mother and child health (MCH) interventions suitable to the local situation, provincial maternal mortality surveillance systems (PMMSS) have been set up in nearly all the provinces in China. Throughout China, the PMMSS is uniform in the basic structure and operation procedure (Figure 1). In China, there is a three-tier system of maternal and child care (MCH) provision and surveillance: county/district, township/street, and village/community. PMMSS uses the MCH institutions network as frontline institutions and personnel for data collection, which is nearly the same with NMCMSS being described in detail in several literatures [1, 2, 5].  Table 1 summarized the main characteristics of PMMSS and provided a comparison with those of two other systems (NMCMSS and NMCHRSS), which are present important data source of maternal mortality in China [1, 2, 5, 6].

However, till now, performances and characteristics of PMMSS in China are not widely known outside of China, since most provincial maternal mortality ratio (PMMR) data were published in Chinese and only five English literatures reported PMMSS and PMMR data in four of 31 provinces [10–14]. The aims of this review were to review the existing papers published in Chinese and English on the current situation of PMMSS and to analyze PMMR in China.

## 2. Methods

### 2.1. Literature Search and Quality Assessment

Searches of publications were performed in the China Academic Journal Network Publishing (CAJNP) database and PubMed. Both searches were limited to literatures reported from January 1982 to May 2013. We searched CAJNP database using keyword combinations of “yun chan fu si wang” (maternal mortality) and “province name” and PubMed using a combination of “maternal mortality or maternal death” and “province name” for all 31 provinces.

Studies were eligible for inclusion if they reported PMMSS and PMMR of any province in China. Studies were excluded if they reported (i) data concerning PMMSS without requisite data (surveillance mode and sampling method) or (ii) data on PMMR only in graphs or percentage.

The quality of included studies was evaluated based on study design and data integrity. A study detailing PMMSS, reporting year-by-year PMMR, cause of maternal death, and results of maternal death review, and analyzing possible factors related to maternal death was considered a high-quality study. A study describing PMMSS in detail and only reporting year-by-year PMMR and cause of maternal death was considered of average quality. Studies that we considered to have unclear or poor design and studies that we judged to present unreliable or conflicting data were regarded to be of poor quality and were excluded from the review.

Two authors independently applied the inclusion criteria (screening) to all identified studies and made decisions on which studies to include.

Two authors did the data extraction independently, and the discrepancies between the authors were resolved by consensus.

### 2.2. Review of PMMSS

Using a classification system developed by the information and statistics centre of the Ministry of Health, which was based on a range of indicators (including the employment rate, the percentage of population under 14 and above 65 years of age, educational achievements and illiteracy rate, crude birth and death rates; infant mortality rate, and gross domestic product (GDP) per capita), China's 31 provinces, autonomous regions, and municipalities were classified into three regions: coast region (high socioeconomic level provinces: Beijing, Fujian, Guangdong, Jiangsu, Liaoning, Shandong, Shanghai, Tianjin, and Zhejiang), inland region (moderate socioeconomic level provinces: Anhui, Chongqing, Guangxi, Hainan, Hebei, Heilongjiang, Henan, Hubei, Hunan, Jiangxi, Jilin, Shaanxi, Shanxi, and Sichuan), and remote region (low socioeconomic level provinces: Gansu, Guizhou, Inner Mongolia, Ningxia, Qinghai, Tibet, Xinjiang, and Yunnan) [15, 16].

The data were extracted from each study that qualified for the inclusion in the review including information about the setup, surveillance mode, sampling percentage, and operation procedures of PMMSS. If two or more studies presented data for the same province in different years, the data were combined.

### 2.3. Pilot Analysis of PMMR Data

The PMMR data (number of live births and maternal deaths by province and by year) were retrieved, pooled, and analyzed. Long and continuous year-by-year PMMR data were prerequisite for the time trend and provincial disparities analysis. However, in the published studies, in most provinces, only discontinuous or short-time PMMR data were available. Six provinces, which had a complete time series of PMMR data, two from coastal region (Shanghai and Liaoning), two from inland region (Henan and Chongqing), and two from remote region (Gansu and Qinghai), were included in the analysis of time trends and provincial disparities on PMMR from 1999 to 2008.

### 2.4. Statistical Methods

Statistical analyses were performed using Stata version 11.0 software (Stata Corp., College Station, TX, USA). Poisson regression was used for the test of time trends and provincial disparities on PMMR. The statistical significance level for *α* was set at 0.05.

## 3. Results

### 3.1. Results of Literature Review

**Figure 2:**
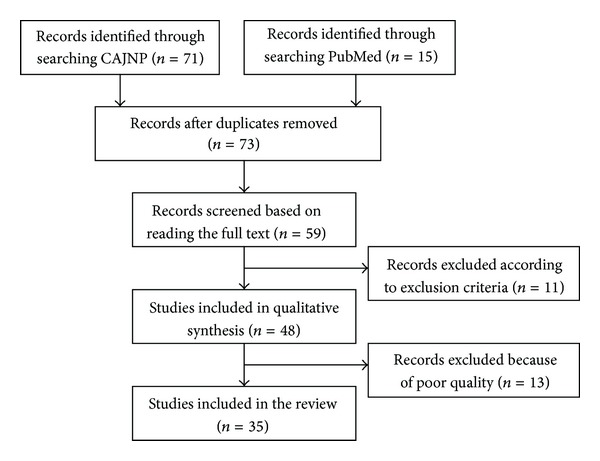
Study selection flow chart.

Figure 2 illustrates the process of literature searching and study selection of the review. A total of 35 studies from 1999 to 2012 were included in the review; 31 of these were reported in Chinese and four in English; 25 of these were published after 2005, and ten of these were published between 1999 and 2003 (Table 2).

On a region level, coast region perform better than the other two regions in terms of data density and integrity on PMMSS and PMMR. Most provinces from coast region had a nearly complete time series of PMMR data from the late 1990s to 2000s; by contrast, most provinces in inland and remote region just reported 3–5 years' PMMR data continuously or intermittently, let alone the five provinces without any data on PMMSS or PMMR (Anhui, Jiangxi, Hebei, and Heilongjiang from inland region and Tibet from remote region) (Table 2).

### 3.2. PMMSS in China

Following the national guide on data collection, maternal death review, and quality control, provincial governments established and adjusted their own PMMSS, respectively, based on their finance resources to monitor local maternal mortality level.

County/district is taken as both the basic unit for data collection and the smallest sampling unit for maternal death surveillance in PMMSS. There were generally two types of surveillance modes for PMMSS: complete maternal death registration and stratified random sampling surveillance. In provinces adopting the former mode, maternal deaths in all counties/districts were reported and audited, whereas, in provinces adopting the latter mode, only the maternal deaths in the sampled counties/districts were under strict monitoring and careful review.

The surveillance mode and the sampling percentage of the PMMSS varied among provinces (Table 3). Of the 26 provinces with available data on PMMSS, 11 provinces (6/9 provinces from coast region, 3/10 provinces from inland region, and 2/7 provinces from remote region) adopted complete maternal deaths registration; the other 15 provinces adopted stratified random sampling surveillance mode, among which Hubei, Liaoning, and Shanxi transferred to complete maternal deaths registration mode in 2001, 2002, and 2006, respectively. The percentage of sampling counties/districts in most provinces adopting stratified random sampling surveillance mode varied from 14.6 to 34.8%, except for the lowest 9.6% in Shaanxi (1996–2000), 40.9% in Ningxia (1996–2002), and the highest 53% in Liaoning (2001).

There was a paucity of data over the time of establishment of PMMSS in most provinces. Only 6 provinces reported the setup times of their PMMSS, which from early to late were Hubei in 1987, Jilin in 1990, Hainan and Henan in 1996, Sichuan in 2001, and Xinjiang in 2004.

### 3.3. The Analysis of the PMMR Data

**Figure 3:**
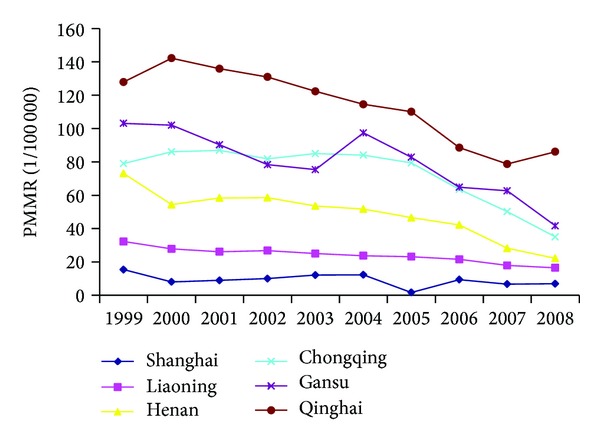
The trend of PMMR in six provinces, 1999–2008.

Figure 3 illustrated the time trends of PMMR in six provinces from 1999 to 2008. Provinces from remote region (Qinghai and Gansu) had the highest risk of maternal mortality, followed by provinces from inland region (Chongqing and Henan) and coast region (Liaoning and Shanghai). PMMR declined significantly in Liaoning, Henan, Chongqing, and Qinghai; the annual RR was 0.94 (95% CI 0.91–0.98), 0.90 (95% CI 0.88–0.92), 0.93 (95% CI 0.92–0.95), and 0.91 (95% CI 0.89–0.94), respectively (all *P* < 0.01). PMMR in Shanghai did not vary significantly from 1999 to 2008 (*P* > 0.05).

Provincial variation in maternal mortality was substantial, especially among the provinces from different regions. Shanghai, with the lowest PMMR, was taken as the reference province. There was no significant difference on PMMR between the two provinces from coast region at all three time spots.

Table 4 showed provincial disparities on PMMR in six provinces in 1999, 2004, and 2008. Shanghai, with the lowest PMMR, was taken as the reference province. No significant difference on PMMR was found between Shanghai and Liaoning at all three time spots (all *P* > 0.05). However, provincial disparities among provinces from different regions were substantial. Impressively, the RR of Qinghai to Shanghai was as high as 8.32 (95% CI 4.07–17.00, *P* < 0.01), 9.36 (95% CI 4.51–19.44, *P* < 0.01), and 7.32 (95% CI 3.11–17.27, *P* < 0.01) in 1999, 2004, and 2008, respectively. Similarly, the RR of Gansu to Shanghai reached up to 6.71 (95% CI 3.32–13.56, *P* < 0.01) and 7.96 (95% CI 3.85–16.47, *P* < 0.01) in 1999 and 2004, respectively.

## 4. Discussion

This is the first English-language review of PMMSS and PMMR in China. With large population coverage and thorough quality control, PMMSS plays an important role in monitoring maternal mortality at provincial level. The surveillance mode and sampling percentage of PMMSS differ widely across the provinces. In addition, wide provincial disparities on PMMR were found after pooled analysis of the PMMSS data from six provinces. The results of the present review will help medical workers, the government, and international organizations understand the current status of provincial level maternal mortality surveillance systems in China.

### 4.1. PMMSS in China

China has begun using NMCMSS from 1989 to track and estimate maternal mortality at national level [1, 2]. However, covering about 5% of the whole population, the national system may not effectively capture the disparities on PMMR among individual provinces [6]. Although major policies remain the same for the entire country of China, the decentralization reform since 1978 enforces the autonomy and initiative of the local government to develop the region's economy and design programs which suit local needs in several aspects including the public health system [17, 18]. In view of the continued prominence of maternal mortality as a health and development goal, local maternal mortality level has been one of the most important indicators for the local governmental performance evaluation [19]. PMMSS have been set up in nearly all the provinces to effectively monitor local PMMR. Following the same national outline for basic structure, data collection, and quality control with NMCMSS in China, which has been accepted as the most reliable data source of China maternal mortality level, a critical assessment of the accuracy of the PMMSS data is warranted.

As a system self-financed by provincial governments, PMMSS are limited to a great degree by the local socioeconomic development level. Consequently, although following the unified national outline for basic structure and data collection, the surveillance mode and sampling percentage of PMMSS differ widely across the provinces. As shown in the results, in the better-off region (coast region), nearly all the provinces realized complete registration of maternal deaths which is certainly effective, but undoubtedly more expensive and logistically challenging, while in inland and remote regions, most provinces adopted stratified random sampling surveillance. However, after experiencing remarkable economic growth, three provinces adjusted their maternal death surveillance mode from sampling surveillance to complete registration of maternal deaths, which also shows clearly the great importance to maternal death surveillance and reduction attached by the provincial governments.

The comparison among PMMSS, NMCMSS, and NMCHRS could easily make clear the superiority of PMMSS in monitoring provincial maternal mortality. To some degree, PMMSS fosters strengths and circumvents weaknesses of the two other systems. On one hand, with the same thorough quality control and careful review of the maternal death with NMCMSS, PMMSS have substantially larger population coverage than NMCMSS. Within the country, PMMSS in 40% provinces adopt complete maternal deaths registrations; in most provinces adopting sampling surveillance mode, the sampling percentage varies from 14.6 to 34.8%, which is obviously higher than the sampling percentage of the NMCMSS (about 5%) [2, 5]. On the other hand, compared to NMCHRS which covers the whole population, PMMSS may be better at capturing maternal deaths due to the active searching for maternal deaths, medical certification of maternal deaths, and thorough quality control. The available PMMR from PMMSS in 2000 is higher than those from NMCHRS in 2000 which was reported by Yanqiu et al. in most provinces for the same period [6], which has exactly proved the aforementioned viewpoint. Moreover, the added strength of the PMMSS is that every maternal death is confirmed and reviewed in detail, which means that the causes of each death are recorded, allowing for learning lessons and determining the actions necessary to reduce MMR. Taken together with large population coverage and thorough quality control, PMMSS may be the most reliable data source for estimation of provincial maternal mortality in each province.

### 4.2. Wide Provincial Disparities on PMMR

Several papers have highlighted the problem of regional disparities on MMR in China. Feng et al. [1] and Liang et al. [5] analyzed the data from NMCMSS and found that MMR went from low to high from the coast region to remote region. Using another data source NMCHRS, Yanqiu et al. [6] drew a similar conclusion that there was significant difference on MMR among different geographical regions. Using the data from PMMSS, our analysis concurred with previous findings suggesting that there were wide provincial disparities on PMMR between better-off and economically more deprived provinces. From 1999 to 2008, PMMR of Shanghai, one of the most prosperous provinces in China, was about 10 per 100,000 live births, comparable to the level of many developed countries. Nevertheless, in the two provinces from remote region, the risk of maternal death reached up to 7–9 times that in Shanghai. Although the data available in this review (only covering six provinces) does not allow further conclusions to be drawn, the review is the first to reveal the disparities on MMR at provincial level. Further study on the related factors of the wide provincial disparities is warranted.

Tibet, the province with the most underdeveloped economies and the highest MMRs around the country [20], definitely merits discussion when it comes to the provincial disparities on PMMR. According to the data of NMCHRS, PMMR in Tibet was 466.9 and 290.3 per 100 000 live births in 2000 and 2005, respectively [6]. However, we found no paper on the PMMSS or PMMR in Tibet; it might reflect that the lag of economic development and maternal heath administrative management lead to the miss of publication or no PMMSS in Tibet. The situation is similar to another province, Xinjiang, from remote region, which has highest PMMR in this review, where the PMMSS had not been set up till 2004 and the PMMR from 2004 to 2006 reached up to 143.2 per 100,000 live births. We venture to suggest that, in the economically most deprived provinces from remote region, central government could do more in assisting the provincial government by providing extra funding and personnel training for PMMSS and in strengthening supervision of local maternal mortality reduction.

Many studies have drawn attention to underreporting of births in national vital registration systems and fertility surveys, and the PMMSS is probably no exception. Underreporting of births is higher in remote areas or provinces, and the missing births are at higher risk of death; then we may have underestimated the maternal mortality ratio in remote areas or provinces and the gap between remote areas or provinces and coast areas or provinces. However, the same biases are likely to apply to maternal deaths over time, so this should not have influenced the observed disparities in the MMR over time.

### 4.3. Limitation of the Review

Several limitations to our review should be acknowledged here. First, there is obvious lack of information from the literatures. In China, maternal mortality surveillance findings usually are regarded as “internal report,” “confidentiality” were applied by restricting “outsider” access to the details [21]. For a long time, the results were only released through 2-3 page publications in Chinese medical journals. Even in the published articles, only gross aggregated findings were reported, and some important issues such as the review results of the maternal death were generically mentioned. Publishing the results of PMMSS in detail, which can serve a pivotal role in identifying the factors contributing to maternal deaths, learning lessons to save lives, and determining the actions necessary to reduce problems, should be encouraged.

Second, generalizing conclusions about the national level MMR or regional level MMR from the PMMSS data has inherent limitation, since the different surveillance modes among the different provinces lead to big differences in the surveillance population, which necessarily hinders the pooled analysis.

## 5. Conclusion

Although it may be ambitious, our conclusion is that PMMSS may be the most reliable data source for measuring provincial level MMR in each province. Given the size and diversity of China, it would be advantageous to keep the national surveillance systems as well as the provincial system. Great provincial disparities on PMMSS and PMMR do exist within China. More emphasis should be placed on improving PMMSS and reducing PMMR, particularly in the provinces with high maternal death burden, in order to ensure that all of the PMMSS are robust and adequate and provide comparable and complete information and that MMR could have a balanced decrease across the whole of China. In addition, the publication and wide spread of PMMSS data should be encouraged.

## Figures and Tables

**Table 1:** Comparisons among PMMSS, NMCMSS, and NMCHRS.

	PMMSS	NMCMSS	NMCHRS
Establishment	Established by the provincial governments at varied time	Established by Ministry of Health (MOH) in 1989 and redesigned in 1996	Established by MOH in 1980s
Purpose	Monitor provincial maternal mortality level	Monitor national maternal mortality level	Provide national MCH indicators*
Finance	Funded by local provincial finance	Funded by China's Ministry of Finance	Funded by China's Ministry of Finance
Methodology	Varied by province (see Table 3)	Population-based stratified cluster random sampling survey	Routine report level by level
Covered population	Cover large population (see Table 3)	Cover about 60 million population (about 5% of China's population)	Cover entire population of mainland China
Data form	Maternal mortality report cards	Maternal mortality report cards	Routine reporting forms for MCH indicators
Maternal death review	Yes	Yes	No
Main usage of the data	Prepared annual provincial maternal and child health report	Prepared annual national maternal and child health report for the MOH	Prepared annual national MCH indicators*

*MCH indicators: MMR, infant mortality rate, neonatal mortality rate, rate of birth defects, and so forth.

**Table 2:** Main characteristics of the studies included in the review.

Province	Authors (reference number)	Year	Language	Study period	Live birth	Maternal death	Average MMR (SD*)	Study quality
Coast region								
Beijing	Shen et al. [22]	2006	Chinese	1995–2004	580857	104	17.9 (5.26)	High
Fujian	Zheng et al. [23]	2001	Chinese	1990–1999	3316092	1527	46.05 (6.11)	High
Guangdong	Tian et al. [24]	2003	Chinese	1992–2000	1851650	602	32.51 (6.71)	High
Guangdong	He et al. [25]	2006	Chinese	2001–2003	462168	105	22.72 (1.36)	High
Jiangsu	Zhou and Yu [26]	1999	Chinese	1990–1997	5375632	2431	45.22 (11.68)	High
Liaoning	Shi et al. [27]	2002	Chinese	1990–2000	869298	414	47.6 (11.62)	Average
Liaoning	Li et al. [28]	2012	Chinese	2001–2010	2703606	476	17.61 (5.72)	High
Shandong	Zhao et al. [29]	2002	Chinese	1990–2001	927656	314	33.85 (11.65)	High
Shanghai	Zhu et al. [10]	2009	English	1996–2005	537669	70	10.23 (5.33)	High
Shanghai	Du et al. [11]	2012	English	2000–2009	680005	55	8.1 (3.06)	High
Tianjin	Wen et al. [30]	2009	Chinese	1997–2006	591866	87	14.7 (5.23)	High
Zhejiang	Qiu et al. [12]	2010	English	1988–2008	8880457	2258	25.43 (10.23)	Average
Inland region								
Chongqing	Mou et al. [31]	2000	Chinese	1986–1997	1675839	1582	94.4 (21.02)	Average
Chongqing	Zhou et al. [32]	2009	Chinese	1999–2008	2590959	1862	71.87 (17.75)	High
Guangxi	Dai et al. [33]	1999	Chinese	2003	203919	145	71.12 (0)	High
Guangxi	Dai et al. [34]	2002	Chinese	2010	297204	55	18.51 (0)	High
Hainan	Chen [35]	2012	Chinese	2001–2010	908814	315	34.66 (8.16)	High
Henan	You et al. [14]	2012	English	1996–2009	2483327	1129	45.46 (19.31)	High
Hubei	Wang [36]	2005	Chinese	1996-1997	151266	102	67.43 (27.85)	High
Hubei	Wang and Ma [37]	2007	Chinese	2001	377036	173	45.88 (0)	High
Hunan	Ding et al. [38]	2005	Chinese	1996–2002	3561124	2032	57.06 (15.43)	High
Jilin**	Yu et al. [39]	2012	Chinese	2004–2009				Average
Shaanxi	Xia et al. [40]	2003	Chinese	1996–2000	121341	77	63.46 (6.88)	Average
Shanxi	Yuan et al. [41]	2008	Chinese	1996–2003	935356	528	56.44 (10.22)	High
Shanxi	Wei et al. [42]	2011	Chinese	2006	285913	111	38.82 (0)	High
Sichuan**	Wu et al. [43]	2011	Chinese	2001–2010				Average
Remote region								
Gansu	Chen et al. [44]	2007	Chinese	1996–2005	673577	721	107.04 (30.73)	High
Gansu**	Ma et al. [45]	2011	Chinese	2005–2010				Average
Guizhou	Ma et al. [46]	2005	Chinese	2000–2004	322083	374	116.12 (11.56)	High
Inner Mongolia	Wang et al. [47]	2007	Chinese	1999–2005	1265554	680	53.73 (9.16)	Average
Ningxia	Liu et al. [48]	2006	Chinese	1996–2002	183428	165	89.95 (26.48)	High
Qinghai	Zhang et al. [49]	2002	Chinese	1999	35966	46	127.9 (0)	High
Qinghai	Zhao [50]	2010	Chinese	2000–2008	579329	619	106.85 (23.15)	Average
Xinjiang	Wang et al. [51]	2009	Chinese	2004–2006	131968	189	143.2 (19.41)	High
Yunnan	Yu et al. [52]	2010	Chinese	2006–2009	1867486	952	51 (9.35)	High

*SD: standard deviation.

**Jilin, Sichuan, and Gansu: although the average MMR of Jilin (2004–2009), Sichuan (2001–2010), and Gansu (2005–2010) could not be calculated due to the lack of the data about number of live births and maternal deaths, we extracted a rough variation tendency of PMMR in these three provinces: for Jilin, decreased from 32.24 in 2004 to 27 and 12 in 2009; for Sichuan, decreased from 66.52 in 2001 to 39.66 in 2010; and for Gansu, decreased from 82.79 in 2005 to 35.39 in 2010.

**Table 3:** The surveillance mode and sampling percentage of PMMSS.

Province	Surveillance mode (study period)	Percentage of sampled counties/districts
Coast region		
Shanghai	Complete registration of maternal deaths (1996–2009)	100%
Tianjin	Complete registration of maternal deaths (1997–2006)	100%
Beijing	Complete registration of maternal deaths (1995–2004)	100%
Jiangsu	Complete registration of maternal deaths (1990–1997)	100%
Fujian	Complete registration of maternal deaths (1990–1999)	100%
Zhejiang	Complete registration of maternal deaths (1988–2008)	100%
Liaoning	Stratified random sampling surveillance (1990–2001) Complete registration of maternal deaths (2002–2010)	1990–2000: 25%; 2001: 53%; 2002–2010: 100%
Guangdong	Stratified random sampling surveillance (1992–2003)	1992–1995: 14.6%; 1996: 34.1%; 1997–2003: 19.5%
Shandong	Stratified random sampling surveillance (1990–2001)	14.80%
Inland region		
Jilin	Complete registration of maternal deaths (2004–2009)	100%
Hunan	Complete registration of maternal deaths (1996–2002)	100%
Chongqing	Complete registration of maternal deaths (1986–1997, 1999–2008)	100%
Shanxi	Stratified random sampling surveillance (1996–2003) Complete registration of maternal deaths (2006)	1996–2003: 33.6%; 2006: 100%
Hubei	Stratified random sampling surveillance (1996-1997) Complete registration of maternal deaths (2001)	1996-1997: 22.4%; 2001: 100%
Guangxi	Stratified random sampling surveillance (2003, 2010)	2003: 26.9%; 2010: 28.2%
Henan	Stratified random sampling surveillance (1996–2009)	23.10%
Sichuan	Stratified random sampling surveillance (2001–2010)	2001–2005: 16.2%; 2006–2010: 26%
Shaanxi	Stratified random sampling surveillance (1996–2000)	9.60%
Hainan	Stratified random sampling surveillance (2001–2010)	Not indicated
Remote region		
Yunnan	Complete registration of maternal deaths (2006–2009)	100%
Inner Mongolia	Complete registration of maternal deaths (1999–2005)	100%
Ningxia	Stratified random sampling surveillance (1996–2002)	40.90%
Gansu	Stratified random sampling surveillance (1996–2010)	34.80%
Qinghai	Stratified random sampling surveillance (1999–2008)	1999–2004: 21.7%; 2005–2008: 28.3%
Guizhou	Stratified random sampling surveillance (2000–2004)	19.20%
Xinjiang	Stratified random sampling surveillance (2004–2006)	15.90%

**Table 4:** Time trend of provincial disparities on MMR in six provinces.

	1999 RR (95% CI)	2004 RR (95% CI)	2008 RR (95% CI)
Coast region			
Shanghai	1	1	1
Liaoning	2.06 (0.94–4.53)	1.48 (0.70–3.14)	2.26 (0.96–5.28)
Inland region			
Henan	4.75 (2.39–9.44)*	4.23 (2.06–8.68)*	3.21 (1.39–7.43)*
Chongqing	5.14 (2.63–10.02)*	6.87 (3.39–13.92)*	5.10 (2.24–11.61)*
Remote region			
Gansu	6.71 (3.32–13.56)*	7.96 (3.85–16.47)*	
Qinghai	8.32 (4.07–17.00)*	9.36 (4.51–19.44)*	7.32 (3.11–17.27)*

*P < 0.01; no requisite data was available for calculate the Gansu to Shanghai RR in 2008.
